# Anticancer activity of the iron facilitator LS081

**DOI:** 10.1186/1756-9966-30-34

**Published:** 2011-03-31

**Authors:** Zhen Li, Hiroki Tanaka, Floyd Galiano, Jonathan Glass

**Affiliations:** 1 Feist-Weiller Cancer Center, Department of Medicine, LSU Health Sciences Center, Shreveport, Louisiana. 1501 Kings Highway, Shreveport, LA 71130, USA

## Abstract

**Background:**

Cancer cells have increased levels of transferrin receptor and lower levels of ferritin, an iron deficient phenotype that has led to the use of iron chelators to further deplete cells of iron and limit cancer cell growth. As cancer cells also have increased reactive oxygen species (ROS) we hypothesized that a contrarian approach of enhancing iron entry would allow for further increased generation of ROS causing oxidative damage and cell death.

**Methods:**

A small molecule library consisting of ~11,000 compounds was screened to identify compounds that stimulated iron-induced quenching of intracellular calcein fluorescence. We verified the iron facilitating properties of the lead compound, LS081, through ^55^Fe uptake and the expression of the iron storage protein, ferritin. LS081-induced iron facilitation was correlated with rates of cancer cell growth inhibition, ROS production, clonogenicity, and hypoxia induced factor (HIF) levels.

**Results:**

Compound LS081 increased ^55^Fe uptake in various cancer cell lines and Caco2 cells, a model system for studying intestinal iron uptake. LS081 also increased the uptake of Fe from transferrin (Tf). LS081 decreased proliferation of the PC-3 prostate cancer cell line in the presence of iron with a lesser effect on normal prostate 267B1 cells. In addition, LS081 markedly decreased HIF-1α and -2α levels in DU-145 prostate cancer cell line and the MDA-MB-231 breast cancer cell lines, stimulated ROS production, and decreased clonogenicity.

**Conclusions:**

We have developed a high through-put screening technique and identified small molecules that stimulate iron uptake both from ferriTf and non-Tf bound iron. These iron facilitator compounds displayed properties suggesting that they may serve as anti-cancer agents.

## Background

Iron is an essential element required for many biological processes from electron transport to ATP production to heme and DNA synthesis with the bulk of the iron being in the hemoglobin of circulating red blood cells [1,2]. Too little iron leads to a variety of pleiotropic effects from iron deficiency anemia to abnormal neurologic development, while too much iron may result in organ damage including hepatic cirrhosis and myocardiopathies. The system for the maintenance of iron homeostasis is complex. Approximately 1 mg of the iron utilized daily for the synthesis of nascent red blood cells is newly absorbed in the intestine to replace the amount lost by shed epithelial cells and normal blood loss. The remainder of the iron incorporated into newly synthesized hemoglobin is derived from macrophages from catabolized senescent red blood cells. Hence, the uptake of iron for its final incorporation into hemoglobin or other ferriproteins requires 3 different transport pathways: intestinal iron absorption, iron release from macrophages, and iron uptake into erythroid precursors and other iron-requiring cells.

In vertebrates, iron entry into the body occurs primarily in the duodenum, where Fe^3+ ^is reduced to the more soluble Fe^2+ ^by a ferrireductase (DcytB), which transports electrons from cytosolic NADPH to extracellular acceptors such as Fe^3+ ^[3]. The Fe^2+ ^is transported across the brush border membrane (BBM) of duodenal enterocytes via the transmembrane protein, DMT1 (divalent metal transporter, also known as SLC11a2, DCT1, or Nramp2) [4,5]. Subsequently, the internalized Fe^2+ ^is transported across the basolateral membrane (BLM) by the transmembrane permease ferroportin (FPN1, also known as SLC40a1) [3,6] in cooperation with the multicopper oxidase Hephaestin (Heph) [7,8]. The exit of iron from macrophages onto plasma transferrin (Tf) is also mediated by the interaction of FPN1 and Heph [9]. The efflux of iron into the systemic circulation from the enterocyte and the macrophage is negatively regulated by hepcidin, the iron-stores regulator. Hepcidin binds to FPN1 promoting phosphorylation, internalization, and subsequent catabolism of FPN1 via proteasomes [10].

In erythroid precursor cells, and indeed in all non-intestinal cells, iron uptake is mediated by receptor mediated endocytosis of ferri-transferrin (Fe-Tf) although routes for non-transferrin bound Fe (NTBI) also exist. Fe-Tf binds to the transferrin receptor (TfR) on the cell surface [11] and the Fe-Tf complex is internalized into endosomes with subsequent acidification of the endosome which releases Fe^3+ ^from Tf. The Fe^3+ ^is then reduced to Fe^2+ ^by the ferrireductase STEAP 3 [12] and the Fe^2+ ^transported by DMT1 into the cytosol.

There are two situations in which one could envision a benefit from being able to accelerate or otherwise increase cellular uptake of iron. First, iron deficiency is endemic in much of the world resulting in decreased ability to work especially in women of child bearing age and in impaired neurologic development in children [13,14]. Common factors leading to an imbalance in iron metabolism include insufficient iron intake and decreased absorption due to poor dietary sources of iron [15]. In fact, Fe deficiency is the most common nutritional deficiency in children and the incidence of iron deficiency among adolescents is also rising [16]. Iron deficiency ultimately leads to anemia, a major public health concern affecting up to a billion people worldwide, with iron deficiency anemia being associated with poorer survival in older adults [17]. As much of iron deficiency is nutritional, drugs that promote iron uptake could be beneficial without the necessity of changing economic and cultural habits that dictate the use of iron poor diets.

A second, and separate, situation exists in malignancies. Cancer cells often have an iron deficient phenotype with increased expression of TfR, DMT1, and/or Dcytb and decreased expression of the iron export proteins FPN1 and Heph [18-20]. Since higher levels of ROS are observed in cancer cells compared to non-cancer cells drugs that stimulate iron uptake into cancer cells might further increase ROS levels via the Fenton reaction. The increased ROS might lead to oxidative damage of DNA, proteins, and lipids [21,22] and cell death or potentiate cell killing by radiation or radiomimetic chemotherapeutic agents. Further, increased intracellular levels of Fe would increase the activity of prolyl hydroxylases potentiating hydroxylation of HIF-1α and HIF-2α, transcription factors that drive cancer growth, resulting in decreased HIF expression via ubiquination and proteasome digestion.

Wessling-Resnick and colleagues have used a cell-based fluorescence assay to identify chemicals in a small molecule chemical library that block iron uptake [23-25]. While some of the chemicals identified inhibited Tf-mediated iron uptake [23] more recent studies utilizing a HEK293T cell line that stably expresses DMT1 have identified chemicals that act specifically on the iron transporter [24,25]. In the current study, we have used a similar assay to identify chemicals that increase iron uptake into cells and demonstrate that these chemicals are effective in increasing iron transport across Caco2 cells, a model system for studying intestinal iron absorption, and increasing iron uptake into various cancer cell lines, favourably altering several aspects of the malignant phenotype.

## Methods

### Cell lines and Chemicals

All antibodies were purchased from Santa Cruz Biotechnology, Inc. (Santa Cruz, CA) except for rabbit anti-HIF-1α and -2α which were purchased from Novos Biologicals (Littleton, CO). All analytical chemicals were from Sigma-Aldrich (St. Louis, MO). The chemical libraries were obtained from ChemDiv (San Diego, CA) and TimTec (Newark, DE). CM-H_2_DCFDA (5-(and-6)-chloromethyl-2',7'-dichlorodihydrofluorescein diacetate, acetyl ester) or DCFDA and calcein-AM were from Invitrogen (Carlsbad, CA). The cell lines K562, PC-3, Caco2, MDA-MB231, and 267B1 were obtained from ATCC (Bethesda, MD). RPMI1640 and DMEM culture media and fetal calf serum (FCS) were obtained from Atlanta Biologicals (Lawrenceville, GA).

### Screening for chemicals that increase iron uptake

K562 cells were loaded with calcein by incubating cells with 0.1 μM of Calcein-AM for 10 min in 0.15 M NaCl-20 mM Hepes buffer, pH 7.4, with 0.1% BSA at 37°C followed by extensive washing with NaCl-Hepes buffer to remove extracellular bound calcein, and aliquoted at 5 × 10^4 ^- 1 × 10^5 ^cells/well in 96-well plates containing test compounds at 10 μM and incubated for 30 min in a humidified 37°C incubator with 5% CO_2 _before baseline fluorescence was obtained at 485/520 nm (excitation/emission) with 0.1% DMSO as the vehicle control and DTPA as a strong iron chelator control to block all iron uptake. The fluorescence was then obtained 30 min after addition of 10 μM ferrous ammonium sulfate in 500 μM ascorbic acid (AA). The percentage of fluorescence quench was calculated relative to 200 μM DTPA added as a blocking control and DMSO as a vehicle control as follows:(1)

where Δ F is the change in fluorescence, or fluorescence quench, observed in any well, F_0 _represents the fluorescence after 30 min of compound, and F_f _represents the fluorescence 30 min after addition of Fe. These results were normalized to the blocking and vehicle controls as follows:(2)

where Δ F_n _is the normalized quench observed after addition of iron, F_compound _is the Δ F observed with compound, F_min _is the average Δ F of the DMSO control; and F_max _is the average Δ F of the DTPA control. With this normalization 100% indicates that a test compound is as potent as DTPA in blocking iron-induced quenching and 0% indicates no inhibition of iron quenching by a test compound or the same quench as observed with the DMSO vehicle control. Compounds with Δ F_n _between 0% and 100% are defined as inhibitors of iron uptake. Negative values for Δ F_n _represent compounds that facilitate iron uptake into cells. Our criteria for active compounds to be further investigated was arbitrarily set as Δ F_n _= 50-100% quenching for iron uptake inhibitors and < -50% quenching for iron uptake facilitators.

### ^55^Fe uptake into K562 cells

3 × 10^5 ^K562 cells in 300 μl NaCl-Hepes-0.1% BSA were incubated for 30 min with test compound at various concentrations as indicated in a humidified 37°C incubator with 5% CO_2_. A mixture of ^55^Fe- and AA was then added for a final concentration of 1 μM ^55^Fe -1 mM AA and the cells incubated for an additional 60 min. The reaction was stopped by the addition of ice-cold quench buffer (NaCl-Hepes with 2 mM EDTA) followed by extensive washing of the cells which were then dispersed in scintillation fluid and ^55^Fe radioactivity determined in a Tri-carb 2900 TR liquid scintillation analyzer (Packard BioScience Company, Meriden, CT).

### Preparation of medium containing 10% FCS with iron-saturated Tf

Iron on the Tf in FCS was removed from the Tf by lowering the pH to 4.5 followed by dialysis against 0.1 M citrate buffer, pH 4.5, in the presence of Chelex for 16 hours, and dialyzed again against HEPES buffered saline, pH 7.4, in the presence of Chelex. FeNTA (1:2 molar ratio for Fe: NTA) was then added to the now iron-free FCS at 1 mM final concentration followed by extensive dialysis against HEPES buffered saline, pH 7.4. The resulted FCS containing iron-saturated Tf was added into RPMI1640 to make the medium containing 10% iron-saturated FCS.

### Western blot analysis of ferritin, TfR, and HIF-1α and -2α

PC-3 cells were plated into 6-well plates at cell density of 5 × 10^5 ^cells/well for overnight attachment before addition of test compound or vehicle control for 16 hours. The cells were then lysed with RIPA buffer (50 mM Tris-HCl, 1% NP-40, 0.25% Na-deoxycholate, 150 mM NaCl, 1 mM EDTA, pH 7.4) and the lysates separated on SDS-PAGE with subsequent transfer to nitrocellulose for western blot analysis using the following antibodies: mouse anti-human ferritin-heavy chain, mouse anti-human TfR, anti-HIF-1α or -2α, and rabbit anti-human β-actin. Results were quantitated by densitometry and relative densitometric units expressed as the ratio of protein of interest to actin.

### ^55^Fe uptake and transport in Caco2 cells

Caco2 cells were seeded in 6.5 mm bicameral chambers in 24-well plates, grown in 10% FCS-minimum essential medium for ~2 week to reach a transepithelial electrical resistance (TEER) of 250 ^.^cm^2^. The cells were incubated in serum-free DMEM with 0.1% BSA overnight and the inserts then transferred to fresh 24-well plates with the basal chambers containing 700 μL of 20 μM Apo-Tf in DMEM. Test compound at concentrations of 0-100 μM in a total volume of 150 μl were added to the top chamber, incubated for 60 min at 37°C, 5% CO_2 _incubator, followed by the addition of ^55^Fe to the top chamber at a final concentration of 0.125 μM ^55^Fe in 1 mM AA. At various times up to 2 hours, the top and bottom chamber buffer were removed, the cell layer washed extensively with Hepes-NaCl containing 0.1 mM EDTA, and ^55^Fe radioactivity determined in the upper and lower chamber buffers and the cell layer.

### ROS measurement

To determine if compound affected cellular production of ROS, 5 × 10^5 ^K562 cells were washed, treated for 30 min with compound in Hepes-NaCl buffer, and intracellular levels of ROS detected with CM-H_2_DCFDA by flow cytometry as described [26]. ROS levels are presented as mean fluorescence intensity in the appropriate gated areas. K562 cells exposed to 10 μM H_2_O_2 _were used as positive control for ROS generation.

### Cell proliferation and colony formation assays

To assess cell proliferation PC-3 cells were seeded into 96-well plates at 1 × 10^4^/well for 24 hr to allow for cell attachment. Cells were treated with 0.1% DMSO, 10 μM ferric ammonium citrate, 10 μM LS081, or the combination of 10 μM Fe + 10 μM LS081 in RPMI1640-10% FCS for 24-72 hr with the treatment media being replenished every 24 hr. Cell proliferation was accessed 24, 48, or 72 hr after treatment. In separate experiments, PC-3 or 267B1 cells were plated in 96-well plates at 1 × 10^4^/well in RPMI1640 containing 10% FCS overnight before 24 hr treatment with 0.1% DMSO, 2 μM ferric ammonium citrate, 3 or 10 μM LS081 ± Fe in serum-free-RPMI1640, with an additional 24 hr incubation in RPMI-1640-10% FCS without LS081. Cell proliferation was assayed with CellTiter 96 AQ_ueous _Non-Radioactive Cell Proliferation Assay (Promega) kit on a Synergy 2 Spectrophotometric Analyzer (BioTek Inc., Winooski, Vermont) with wavelength of 490 nM and the results standardized to the percentage of inhibition induced by DMSO alone. Cell viability was assessed by Trypan blue exclusion.

Colony formation was assayed in PC-3 cells by plating 500 cells/well in 6-well plates in 10% FCS-RPMI1640 for 48 hr, followed by incubation with 0.1% DMSO, 10 μM ferric ammonium citrate, 3 or 10 μM LS081 ± ferric ammonium citrate for an additional 48 hours, after which the media was replaced with 10% FCS-RPMI1640. The cells were cultured for an additional 10-14 days and then stained with Crystal violet before colonies consisting of more than 50 cells were enumerated.

## Results

### A cell based fluorescence assay to screen small molecules that increase iron transport into cells

**Figure 1:**
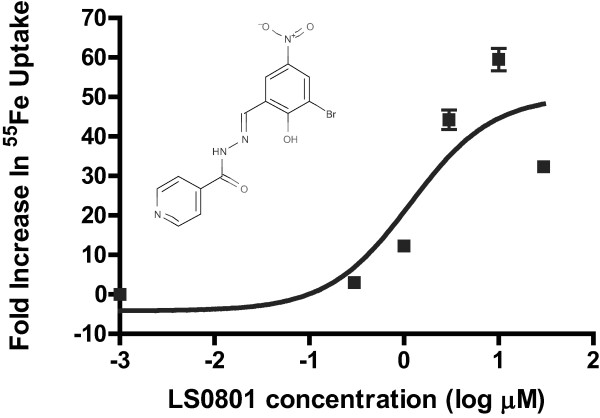
**Dose response curve of LS081 on ^55^Fe uptake in K562 cells**. ^55^Fe uptake was measured as described in the Methods. Briefly, 3 × 10^5 ^K562 cells were incubated with LS081 for 30 min at concentrations of 0.1-100 μM prior to the addition of 1 μM ^55^Fe-1 mM AA with subsequent determination of intracellular ^55^Fe radioactivity. Results were expressed as fold increase in ^55^Fe radioactivity relative to cells treated with 0.1% DMSO alone. Shown are the means ± SEM of 3 separate experiments with triplicates for each experiment. The insert shows the chemical structure of LS081.

We utilized an intracellular calcein fluorescence screening method modified from Brown *et al. *[23] to screen a library consisting of ~11000 small molecules for their ability to increase or decrease iron uptake into cells. As noted in the Method, compounds which enhanced the calcein fluorescence quenching induced by iron were considered to be iron facilitators while those that decreased fluorescence quenching were considered inhibitors of iron uptake. In the initial screening of the compounds obtained from ChemDiv thirty compounds exhibited negative values for Δ F_n_, i.e. Δ F_n _< -50% and were therefore defined as iron facilitators including a number of hydrazone compounds. A similar number of compounds had Δ F_n _= 50-100% and were defined as iron uptake inhibitors. About 10 of these inhibitors blocked the *in vitro *quenching of calcein by iron and were therefore presumably iron chelators. An additional 80 structural analogs of the hydrazone class of facilitators obtained from TimTec were subsequently assessed with 16 more facilitators identified. The ability to facilitate iron uptake was verified using a dose response curve from 0.1 - 100 μM of a putative facilitator with the same calcein quenching assay as well as by measuring the effect of the presumed facilitators on ^55^Fe uptake into K562 cells. Additionally, we arbitrarily chose as the lead compound LS081, the first compound to be verified by a dose-response curve (Figure 1). The ability to facilitate iron uptake was confirmed by dose response curves in 14 of the 16 facilitators identified on the initial screen. The EC_50 _for LS081 was 1.22 ± 0.48 μM with a range of EC_50 _of 0.5-2 μM for the remainder of the iron facilitators. Within the range of concentrations used over the length of the screening neither cell number nor cell viability was affected; in addition, the chemicals did not affect the *in vitro *quenching of calcein by iron (data not shown).

Caco2 cells grown in bicameral chambers for 2-3 weeks to reach the desired trans-epithelial electrical resistance were used as a model for intestinal iron absorption. Under these conditions the Caco2 cells differentiate to form a confluent, polarized monolayer with the brush border membrane of the apical surface in contact with the buffer of the top chamber which then mimics the intestinal lumen and the basal layer in contact with the bottom chamber which represents the systemic circulation. This model allows assaying in the presence of LS081 the transport of ^55^Fe from the apical chamber into the cells and then into the bottom chamber. In this model over 2 hours, LS081 increased ^55^Fe uptake into the Caco2 cells and into the basal chamber by 4.0 ± 0.66 and 3.71 ± 0.29 fold, respectively, compared to the DMSO-treated control (mean fold change ± SEM of 3 experiments) with P < 0.001 for both uptake and transport into the basal chamber.

### Effect of the iron facilitator LS081 on intracellular levels of ferritin

**Figure 2:**
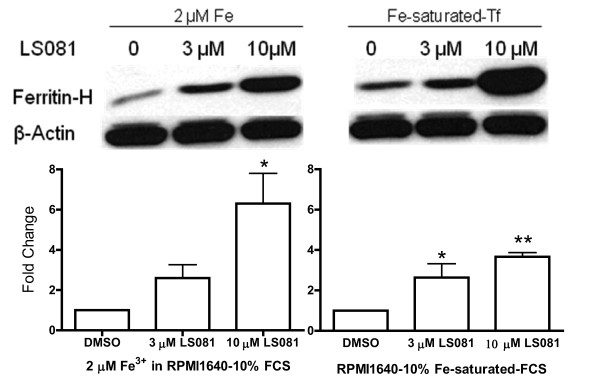
**The effect of LS081 on ferritin expression**. PC-3 cells were treated for 16 hr with DMSO alone, or 3 or 10 μM LS081 in the presence of non-transferrin-bound-iron (ferric ammonium citrate, left panel) or transferrin-bound-iron (Fe-saturated-Tf, right panel). The cellular proteins were separated by SDS-PAGE, and ferritin heavy chain, and β-actin detected by Western blotting as described in the Methods. The top panel shows a representative autoradiography. The bottom panel shows the ratio of ferritin to the actin loading control by densitometric analysis (mean values ± SEM of 3-4 separate experiments). *: p < 0.05, **: p < 0.01 compared to DMSO alone by 1-way ANOVA with Tukey's posttests.

To determine if the increased intracellular iron entered into a metabolically active pool of iron, cellular ferritin levels were measured in PC-3 cells at various times after the addition of LS081. The effects of LS081 on ferritin expression were determined under two conditions: RPMI1640-10% FCS to which 2 μM ferric ammonium citrate was added or RPMI with 10% iron saturated FCS. As shown in Figure 2, LS081 at 3 and 10 μM stimulated ferritin synthesis from both ferric ammonium citrate and iron saturated Tf. In preliminary experiments the level of ferritin protein was not significantly increased by compound alone (data not shown).

### Iron facilitation is cytotoxic to cancer cells

**Figure 3:**
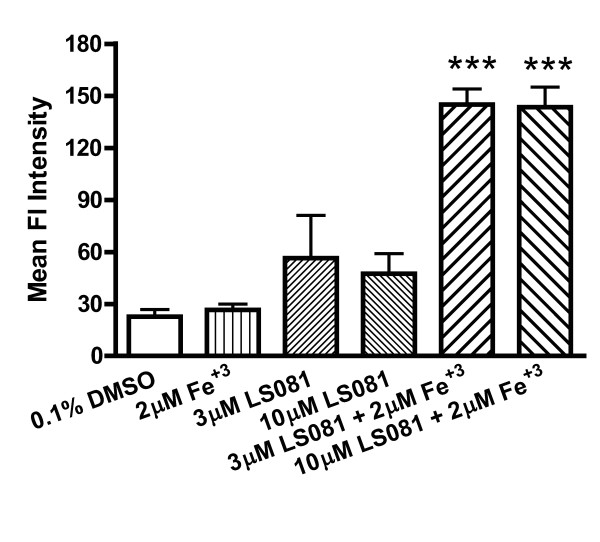
**The effect of LS081 on ROS generation**. Approximately 5 × 10^5 ^K562 cells were treated for 30 min with 0.1% DMSO alone, 10 μM ferric ammonium citrate alone, 3 or 10 μM LS081 alone, or the combination of Fe and LS081 at the indicated concentrations. The cells were then incubated with DCFDA and fluorescence measured by a BD Calibur Flow cytometer expressing the fluorescence as the mean total fluorescence intensity in the gated area. Shown are the means ± SEM of 3 separate experiments with 2-3 replicates for each experiment. *** denotes P < 0.001 compared to the DMSO, Fe, or LS081 alone by 1-way ANOVA with Tukey's posttests.

We examined the effect of the iron facilitator LS081 on ROS generation using DCFDA whose fluorescence intensity is increased in response to elevated intracellular ROS. As shown in Figure 3, K562 cells had significantly increased levels of ROS production when exposed to LS081 in the presence of ferric ammonium citrate but not with iron or LS081 alone.

**Figure 4:**
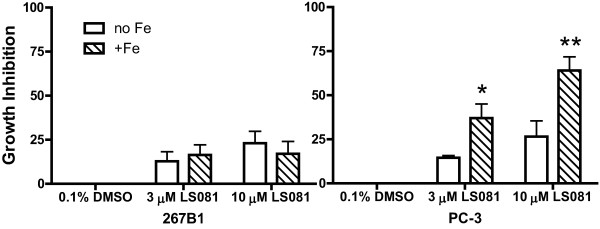
**Effect of LS081 on the proliferation of the prostate cancer cells and non-malignant prostate cells**. Both prostate cancer cell line PC-3 and the immortalized, non-malignant prostate cell line 267B1 cells grown in serum-free RPMI1640 with 0.1% bovine serum albumin were treated with 0.1% DMSO or with 3 or 10 μM LS081 ± 2 μM ferric ammonium citrate for 24 hr followed by an additional 24 hr in RPMI1640-10% FCS before cell proliferation was assayed by MTS. The results are expressed as growth inhibition relative to the DMSO controls (means ± SEM of 3-4 independent observations with four replicates in each observation). *: P < 0.05, **: P < 0.01 comparing with or without Fe conditions by 2-way ANOVA with Bonferroni's posttests.

The proliferation of PC-3 cells, a prostate cancer cell line, was not inhibited by 10 μM ferric ammonium citrate or 10 μM LS081 when cultured in 10% FCS-RPMI1640 for 24 or 48 hrs (Table 1) or 72 hr (data not shown). However, as also shown in Table 1, treatment with 10 μM LS081 plus 10 μM ferric ammonium citrate for 24 hr or 48 hr significantly reduced the number of cells relative to controls. When grown in serum-free medium (Figure 4), 267B1 cells, an immortalized, non-malignant prostate cell line, showed slight growth inhibition with 3 or 10 μM LS081 alone with no potentiation of growth inhibition by the addition of 2 μM ferric ammonium citrate. In contrast, when PC-3 cells were grown in serum-free medium, growth inhibition was far greater for the combination of 2 μM ferric ammonium citrate with either 3 μM LS081 (36 ± 6% inhibition) or 10 μM LS081 (64 ± 8% inhibition) compared to LS081 alone (14 ± 1% or 37 ± 8% inhibition for 3 or 10 μM, respectively) (Figure 4, n = 3 experiments). 2 μM ferric ammonium citrate alone did not affect cell proliferation compared to vehicle control (data not shown).

**Table 1:** The effect of LS081 and iron on the proliferation of PC-3 cells

Treatment	24 hours	48 hours
DMSO	1.00 ± 0.00*	1.00 ± 0.00*
10 μM Fe	1.13 ± 0.04***	1.02 ± 0.06*
10 μM LS081	1.05 ± 0.05**	1.01 ± 0.03*
10 μM Fe and LS081	0.81 ± 0.01	0.80 ± 0.09

PC-3 cells at a density of 1 × 10^4 ^in RPMI1640-10% FCS were seeded into 96-well plates for 24 hrs prior to the addition of 0.1% DMSO ± 10 μM ferric ammonium citrate or 10 μM LS081 ± 10 μM ferric ammonium citrate. Cell proliferation was assayed at 24 or 48 hrs after treatments as described in the Methods and the fold-change calculated compared to DMSO alone. Presented are the means of the fold change ± SEM of 3 independent experiments with each experiment performed in 3-4 replicates. * indicates P < 0.05, ** P < 0.01, *** P < 0.001 compared to Fe plus LS081 by 2-way ANOVA with Bonferroni's posttests.

### Effect of the iron facilitator LS081 on clonogenic potential on prostate cancer cells

**Figure 5:**
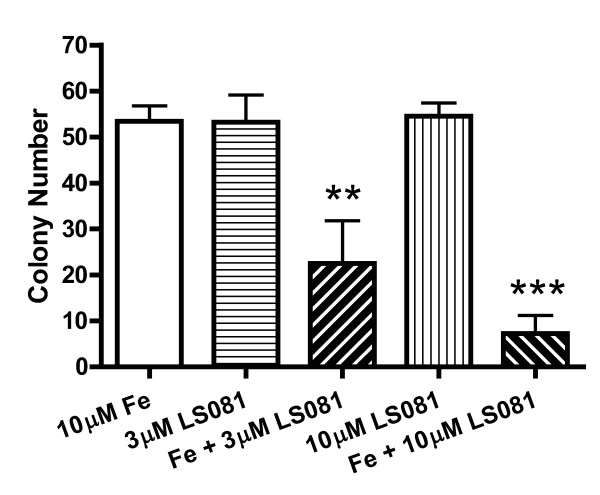
**The effect of LS081 on colony formation of PC3 Cells**. PC-3 cells in 10% FCS-RPMI1640 were seeded at a density of 500 cells/well into 6-well plates. After 24 hrs, cells were treated with 0.1% DMSO, 3 or 10 μM LS081 ± 10 μM ferric ammonium citrate for 48 hrs. The medium was replaced with 10% FCS-RPMI1640 and the cells were allowed to grow for ~ 10-14 days before Crystal violet staining and counting of colonies. Shown are the mean numbers of colonies ± SEM of 3-4 of independent observations with duplicates or triplicates for each observation. **: P < 0.01 compared to either Fe alone or 3 μM LS081 alone; ***: p < 0.001 compared to Fe alone or 10 μM LS081 alone by 1-way ANOVA with Newman-Keuls's posttests.

To determine the effect of LS081 on the clonogenic potential of prostate cancer cells colony formation assays were performed on PC-3 cells in the presence of ferric ammonium citrate in RPMI1640 supplemented with 10% FCS (Figure 5). In combination with iron, LS081 at concentrations of 3 or 10 μM significantly reduced the number of colonies compared to that treated with iron alone or LS081 alone. Reduced colony formation by the combination of Fe and LS081 were also seen in another prostate cancer cell line, DU145, compared to Fe alone (data not shown).

### Effect of the iron facilitator LS081 on the level of HIF-1α and -2α protein

**Figure 6:**
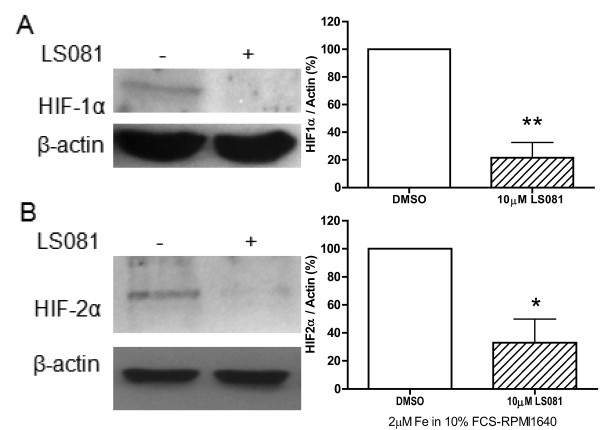
**The effect of LS081 on the expression of HIF1α and HIF2α**. MDA-MB231 and DU145 cells were treated with 10 μM LS081 in 10% FCS-RPMI1640 ± 2 μM ferric ammonium citrate for 16 hr before harvesting for Western blot detection of HIF-1α and 2α as described in the Methods. The Western blots were quantitated by densitometry and the amounts of HIF as the ratio of HIF-1α or HIF-2α to the actin loading control were expressed relative to the DMSO control. The left panels are representative Western blots. A, HIF-1α was detected in DU145 cells cultured at 1% oxygen concentration (hypoxic). In B, HIF-2α was detected in MDA-MB231 cells grown in normal oxygen tension (21%). The right panels show the reduction of HIF-1α or -2α in the treated cells compared to control (means ± SEM of 3-4 experiments). *: p < 0.05; **: P < 0.01 compared to DMSO by 1-way ANOVA with Tukey's posttests.

We investigated if the iron facilitating compound LS081 would affect the level of the transcription factors HIF-1α and -2α. Because the level of HIF-1α in PC-3 cells was too low to be detected by Western blot analysis, especially when cultured at normal oxygen concentrations, we used the prostate cancer cell line DU145 cultured in 1% oxygen as this cell line expressed levels of HIF-1α that could be detected by Western blot analysis. LS081 plus Fe significantly reduced the level of HIF-1α in DU 145 cells (Figure 6A). The effect of LS081 on the level of HIF-2α was also examined using breast cancer cell line MDA-MB-231, because the levels of HIF-2α were too low in prostate cancer cell lines to be detected by Western blot analysis. LS081 significantly reduced HIF-2α expression in MDA-MB-231 cells cultured under normoxic conditions in medium containing 10% FCS (Figure 6B).

## Discussion

As noted by Wessling-Resnik and colleagues in their search for iron uptake inhibitors chemical genetics, i.e. the use of small molecules to perturb a physiologic system, has the ability to shed light on mechanisms of the pathway that is being disturbed [25]. Additionally, compounds that perturb iron uptake could have beneficial, medicinal effects. For example, small molecules which stimulate iron absorption might be used as adjuncts to diets that are iron-deficient. Conversely, molecules that blocked iron uptake might counter the increased iron absorption and resultant iron toxicity often seen in widely prevalent diseases such as sickle cell disease and the thalassemias. Wessling-Resnik has screened chemical libraries to identify chemicals that block iron uptake [23] but also found "activators" of iron uptake which were postulated to have potential as agents to relieve iron deficiency. In the current study we have adapted their calcein-based cell assay and identified compounds that increase iron uptake into Caco2 cells, as a model system for intestinal transport, and into various cancer cell lines, thereby altering several aspects of the malignant phenotype.

In our assay, intracellular calcein fluorescence in K562 cells was quenched upon extracellular iron being transported into the cells. Iron facilitation was defined as fluorescence quenching greater in the presence of a test compound compared to vehicle control. In addition, none of the facilitators appeared to be iron chelators as the chemicals did not compete with iron for calcein quenching in an *in vitro *assay and the iron facilitators affected the cell cycle differently from the iron chelator deferoxamine (data not shown). We did, however, find a number of chemicals that inhibited iron uptake and several of these chemicals appeared to be iron chelators by an *in vitro *assay. Notwithstanding that the faciltators inhibited cell proliferation there was no evidence that the chemicals caused cell lysis as cell number was not diminished during the screening assays or during subsequent measurements of ^55^Fe uptake.

In iron uptake whether from NTBI, in the case of enterocytes, or from ferri-Tf, in the case of all other cell types, the uptake occurs by iron being transported through DMT1. The facilitators could act by activating DMT1, repositioning DMT1 within the cell to more efficiently transport iron, or activating another transporter. DMT1 is a highly insoluble membrane protein making it difficult to determine the effect of the facilitators on DMT1 transport activity in an *in vitro *system; however, a clue to the mode of action of the facilitators comes from our observation that LS081 increased iron uptake when the sole source of iron was ferri-Tf. Iron uptake from Tf requires that the Tf undergo receptor mediated endocytosis and DMT1 is part of the internalized endosome. Hence, for more iron to be delivered to a cell by ferri-Tf the endosomes containing DMT1 must cycle into and out of the cell more rapidly. When iron is delivered by ferri-Tf the rate limiting step in iron uptake is the length of the transferrin cycle, that is the time for ferri-Tf to undergo endocytosis, release iron from Tf into the endosome, and for the now apo-Tf still bound to the TfR to undergo exocytosis and be released from the TfR at the cell surface. If the facilitator shortened the length of the Tf cycle then DMT1 would be internalized more rapidly and the iron from Tf could be delivered faster. Inhibitors of iron uptake from ferri-Tf have been shown to adversely affect the Tf cycle [27]. In enterocytes we and others have shown that DMT1 is internalized upon exposure of the duodenum and Caco2 cells to Fe. Hence, increasing the rate of DMT1 internalization would also increase iron uptake in the enterocytes.

While we presume that LS081 acts via DMT1 by altering the kinetics of DMT1 internalization there are other routes for iron uptake that could be affected. For example, lipocalin (also known as NGAL or 24p3), the L-type Ca^2+ ^channel, and Zip14, a member of zinc transporter family, all have been demonstrated to be iron transporters or channels [28-30]. Whether these potential routes of iron entry are affected by the iron facilitators is not known but these alternative minor routes for iron transport function with NTBI and not with ferri-Tf and could not explain, therefore, how the facilitators affect uptake from ferri-Tf.

Whatever the mechanism(s) by which iron uptake facilitation occurs the Fe that gains entry to the cell enters a pool of metabolically active iron as evidenced by several observations. First, cellular ferritin levels increased in the presence of LS081 whether iron was offered as non-Tf or Tf-bound iron. Second, HIF1α and 2α protein expression was decreased. Third, the colony forming ability of prostate cancer cell lines was decreased. Fourth, LS081 increased the level of ROS.

It is interesting to consider the effects of iron facilitation on the levels of ROS as a possible explanation for the decreased cell proliferation and clonogenicity we observed in cancer cells. ROS levels are increased in cancer cells and it is possible that the additional ROS generation by LS081 exceeds cellular defences. Elevated ROS might then make LS081 treated cells more sensitive to radiation therapy and radiomimetic drugs, a hypothesis that is being actively pursued. The idea of disturbing the redox balance in cancer cells as a therapeutic approach for cancer has been postulated by other investigators [31-33]. Some conventional chemotherapy agents such as melphalan, cisplatin, anthracyclines, or bleomycin, are known to increase ROS by compromising the ROS scavenging capability of cancer cells [34-36]. Dicholoracetate, an inhibitor of pyruvate dehydrogenase kinase, stimulates ROS production and elicits apoptosis in cancer but not in normal cells [37]. Moreover, reducing ROS scavengers by inhibition of glutamate-cysteine ligase, the rate limiting enzyme in glutathione synthesis, increases radiosensitivity of cancer cells [38]. In addition, metal-binding compounds have been considered to be potential anti-cancer agents and have demonstrated anticancer activity [39]. Although some compounds appear to act via metal chelation, others appear to increase intracellular metal concentrations, suggesting different mechanisms of action. For example, clioquinol induces apoptosis of prostate cancer cells by increasing intracellular zinc levels [40], and the anti-malarial drug artemisinin has anti-cancer activity that may be mediated by Fe^2+ ^and/or heme [41,42]. The potential toxicity of excess of iron in cancer cells suggests the benefit of identifying molecules that promote iron uptake into cancer cells triggering more efficient cell death.

Hypoxia is a common feature of most solid tumors with concomitant increased expression of the HIF-1α or HIF-2α components of the HIF transcription factor [43,44]. Elevated levels of HIF-1α or HIF-2α are poor prognostic indicators in a variety of tumors [45]. Under normoxic conditions, both HIF-1α and -2α are hydroxylated by an iron-dependent prolyl hydroxylase (PHD), which requires a ferrous ion at the active site, with subsequent hydroxylation ubiquitination by the von Hipple-Lindau tumor suppressor (VHL) and then proteasome degradation. Higher levels of intracellular iron could facilitate hydroxylation leading to increased ubiquitization and subsequent proteosome degradation of HIF-1α and -2α. HIF expression is important in cancer growth via several mechanisms including neo-vascularization. While HIF-1α and -2α have been targets for drug development [46,47] there is as yet no clinically active drug that specifically targets HIF expression. Presumably LS081 induced reduction in HIF-1α and -2α is directly related to iron facilitation with increased activity of PHD from increased cellular iron, an hypothesis supported by loss of PHD activity and HIF1α stabilization when cellular Fe uptake is limited by TfR knockdown [48].

## Conclusions

In summary, we identified a series of compounds capable of increasing iron uptake into cells. The lead compound, LS081, facilitated iron uptake which resulted in reduced cancer cell growth, colony formation, and decreased HIF-1α and -2α protein levels, suggests that this class of compounds could be a useful anti-cancer agent. In addition, the ability of these compounds to affect iron uptake in a model system of intestinal iron absorption suggests, also, that these compounds have a more general clinical utility for the management of iron deficiency.

## Competing interests

The authors declare that they have no competing interests.

## Authors' contributions

ZL developed the screening techniques, designed and performed most of the experiments and drafted the manuscript. HT performed and analysed part of the screening validation experiments. FG engaged in data acquisition of primary screening. JG developed the strategy to screen for iron regulatory compounds and was involved in data analysis and manuscript revision. All authors read and approved the final manuscript.
